# Different treatments for Crohn’s disease complicated by severe acute lower gastrointestinal bleeding: infliximab therapy is critical and cannot be ignored

**DOI:** 10.3389/fphar.2026.1687439

**Published:** 2026-02-23

**Authors:** Shuoyi Yao, Zheyu Wang, Mingyan Xie, Shengnan Wu, Yingjuan Feng, Xiaoming Liu, Fen Wang

**Affiliations:** 1 Department of Gastroenterology, The Third Xiangya Hospital, Central South University, Changsha, Hunan, China; 2 Hunan Key Laboratory of Non-Resolving Inflammation and Cancer, Central South University, Changsha, Hunan, China; 3 Department of Gastroenterology, Changde Hospital, Xiangya School of Medicine, Central South University (The First People’s Hospital of Changde City), Changde, Hunan, China; 4 Department of Gastroenterology, Yueyang Central Hospital, Yueyang, Hunan, China; 5 Department of Gastroenterology, The Second Affiliated Hospital, Hengyang Medical School, University of South China, Hengyang, Hunan, China

**Keywords:** Crohn’s disease, hemostatic therapy, infliximab, lower gastrointestinal bleeding, surgery

## Abstract

**Objective:**

Acute severe lower gastrointestinal bleeding (SLGIB) is one of the life-threatening complications of Crohn’s disease (CD) whose therapy is being optimized constantly. We aim to evaluate the therapeutic efficacies and economic benefits of different treatments for acute SLGIB in CD.

**Method:**

A multicenter retrospective cohort study was conducted in Hunan Province of China on CD patients with acute SLGIB; here, we analyzed the clinical (hemostatic effects, hemoglobin improvement, rebleeding risk, anti-inflammatory influence, and complications) and economic (duration and cost of hospital stay) characteristics of infliximab, surgical, and traditional hemostatic therapies.

**Results:**

All three groups showed no obvious signs of bleeding in the first week. The negative conversion rates of C-reactive protein (CRP) and erythrocyte sedimentation rate (ESR) in the infliximab therapy group were significantly higher than those in the other two groups (adjusted-*p* < 0.05 for both CRP and ESR), while the increase in hemoglobin did not differ significantly among the three groups (*p* = 0.298). The incidence of post-treatment complications was significantly higher in the surgery (resection) group than the other two groups (adjusted-*p* < 0.05). Cumulative rebleeding risk was lowest in the infliximab therapy group (*p* = 0.001 vs. surgery and *p* = 0.032 vs. traditional therapy). The multivariate COX regression also revealed that surgery [hazard ratio (HR) = 7.270, 95% confidence interval (CI): (1.574, 33.592), *p* = 0.011] and traditional therapy [HR = 4.395, 95% CI: (1.011, 19.113), *p* = 0.048] were independently related to higher rebleeding risk than infliximab therapy. The duration and cost of hospital stay of the infliximab therapy group were significantly lower than those of the surgery group (adjusted-*p* < 0.05) and similar to those of the traditional therapy group (adjusted-*p* > 0.05).

**Conclusion:**

Compared to surgery and traditional therapy (such as somatostatins or octreotide), infliximab therapy could control acute SLGIB in CD as well as achieve similar improvement in hemoglobin level with additional anti-inflammatory effects and lower rebleeding risk. Furthermore, infliximab therapy was found to be more economical than surgery.

## Introduction

Crohn’s disease (CD) is an intestinal idiopathic chronic immune-related inflammatory disease that has higher prevalence in western countries (Cassol et al., 2022; Omede et al., 2025). It is reported that the prevalence of CD in the United States increased from 56 per 100,000 persons in 2010 to 165 per 100,000 persons by 2019 (Hutfless et al., 2024). Furthermore, reasons like changes in the dietary customs have increased the prevalence of CD Asia, South America, and Africa (Cassol et al., 2022; Omede et al., 2025; Park and Cheon, 2021; Balderramo et al., 2021). The incidence of acute severe lower gastrointestinal bleeding (SLGIB) in CD ranges from 0.6% to 6% (Lee et al., 2018; Tu et al., 2024), but the associated mortality rate could be as high as 14.3% (Tu et al., 2024) along with high rebleeding risk (19.0%–50.5%) (Lee et al., 2018; Tu et al., 2024). Hence, acute SLGIB is a serious life-threatening complication of CD whose treatment is being optimized constantly. When acute SLGIB occurs in CD patients, they can choose the conventional hemostatic therapy, but approximately 4.3%–7.6% of patients opt for surgery given the ineffectiveness of medications (Tu et al., 2024). A study by Kim et al. (2012) showed that even under standard treatment, the cumulative rebleeding rate in CD was 33.0% after 1 year and reached almost 72.3% after 10 years. In the treatment of CD, aside from clinical efficacy, we need to carefully consider factors like complications, recurrence, as well as duration and expenses associated with hospitalization (Sands et al., 2024).

Nowadays, the available treatment options for CD include biologics like infliximab, adalimumab, and ustekinumab (Baumgart and Le Berre, 2021), which have exhibited promising efficacy in controlling the progression of CD. In a network meta-analysis, infliximab showed the best efficacy at inducing clinical remission among all agents (Attauabi et al., 2024). According to another meta-analysis based on model stimulation, 44.44% of patients using infliximab achieved clinical remission, while the placebo effect was approximately 21.26%. The clinical response rate of infliximab was reportedly 67.49%, which is the highest, followed by adalimumab (60.34%), with the placebo effect achieving a response rate of 39.48% (Yu et al., 2022). Treatment options using biologics could also reduce the need for surgery in CD patients (Jenkinson et al., 2020). Despite infliximab showing the best efficacy among different biologic agents, very few studies have evaluated the efficacy of infliximab treatment for CD complicated by acute SLGIB, and none of these works have focused on the economic perspective. Therefore, we retrospectively analyzed the therapeutic efficacies and economic benefits of infliximab, surgical, and conventional hemostatic treatments to fill this gap in literature through a multicenter study conducted in Hunan Province of China.

## Methods

### Patient population

Our multicenter retrospective cohort study was performed using data collected from CD patients with acute SLGIB who received infliximab treatment, surgery (resection of lesion parts), or conventional hemostatic therapy (such as somatostatins or octreotide) (Tamagno et al., 2004; Bon et al., 2012; Lavy and Yasin, 2003) between January 2012 and June 2025 at the Third Xiangya Hospital of Central South University, First People’s Hospital of Changde City, Yueyang Central Hospital, and Second Affiliated Hospital of University of South China. This study was conducted in accordance with the principles of the Declaration of Helsinki and was approved by the Clinical Research Ethics Committee of the Third Xiangya Hospital of Central South University (no. I 22153). Owing to the retrospective nature of the study, the Clinical Research Ethics Committee of the Third Xiangya Hospital of Central South University waived the need to obtain informed consent from the participants.

The diagnosis of CD was confirmed through the symptoms, the inflammatory indicators, imaging, endoscopy, and pathology. As noted by Yoon et al. (2021), acute SLGIB was defined as an acute overt lower gastrointestinal bleeding that resulted in (i) an abrupt decrease in hemoglobin (Hb) level to below 9 g/dL or at least 2 g/dL below the baseline, and/or (ii) transfusion of at least two units of blood within 24 h. Patients were excluded from the study if their bleeding incidence was in the upper gastrointestinal tract or caused by hemorrhoids or if they did not meet the criteria for diagnosis of acute SLGIB.

### Data collection

The patient medical records were reviewed retrospectively, and the following data were extracted: gender, date of bleeding, age at the time of first bleeding, Montreal classification of CD, disease duration of CD at the time of bleeding, Crohn’s Disease Activity Index (CDAI) value, smoking history, information on blood transfusion, Hb level, C-reactive protein (CRP) and erythrocyte sedimentation rate (ESR) values before/after the treatment of bleeding, medical treatments of CD before/after bleeding, albumin level at the time of discharge after first bleeding, treatment of bleeding, complications after bleeding treatment, duration of hospitalization, and hospitalization costs. All patients were followed up to record the hemostatic effects and rebleeding rates. The bleeding site was defined as the exact bleeding locus detected by colonoscopy, radionuclide bleeding scan, or angiography (including computed tomography angiography). The treatment was considered successful if there were no obvious signs of bleeding during the time of discharge or the fecal occult blood test result was negative. As per Kim et al. (2021), rebleeding was defined as the occurrence of overt bleeding that satisfied the criteria for acute SLGIB after being discharged.

### Statistical analyses

The median and interquartile range (IQR) values were calculated to describe non-normal continuous variables, whereas normal variables were expressed using the mean and standard deviation (SD). The chi-squared test or Fisher’s exact test was used for categorical variables, and the Kruskal–Wallis test (for non-normal variables) or ANOVA (for normal variables) was used for continuous variables to compare the demographic and clinical characteristics of the three groups. Risk of rebleeding was analyzed using the Kaplan–Meier method. To explore the possible factors related to rebleeding, we performed univariate and multivariate COX regressions, where variables with *p*-values < 0.1 in the univariate analysis were included in the multivariate analysis. We also calculated the hazard ratios (HRs) and corresponding 95% confidence intervals (CIs), with *p*-values < 0.05 being considered as statistically significant. All statistical analyses were performed using SPSS 26.0 software.

## Results

### Baseline characteristics of the patients

A total of 88 CD patients were included in this study who experienced acute SLGIB for the first time; their median follow-up time was 646.5 days, and the patients were grouped into infliximab treatment (n = 24), surgery (n = 19), and traditional treatment (n = 45) groups. Most of the patients in the three groups were male, and the mean age of the infliximab group was lower than those of the other two groups. The ileum was the most common location for CD, and the bleeding sites of the patients were consistent with the CD lesion parts. There was no significant difference among the three groups in terms of baseline Hb, CRP, and albumin levels. The distribution of localized or diffused bleeding among the three groups was not significantly different. The traditional therapy group had the lowest baseline ESR and highest CDAI values among the groups. The detailed baseline characteristics of the patients are listed in Table 1.

**TABLE 1:** Baseline characteristics of the patients included in this study.

Characteristic	Infliximab therapy group (n = 24)	Surgery group (n = 19)	Traditional therapy group (n = 45)	*p-*value
Male sex, %	75.0	78.9	91.1	0.180
Age (SD), years	25.54^a^ (8.53)	38.95^b^ (16.84)	32.22^b^ (10.74)	**0.003**
Active smoker, %	8.3^a^	42.1^b^	22.2^a,b^	**0.032**
Montreal classification, %				
A1	16.7^a^	15.8^a^	13.3^a^	**0.038**
A2	83.3^a^	52.6^a^	66.7^a^	​
A3	0.0^a^	31.6^b^	20.0^a,b^	​
B1	50.0	31.6	51.1	0.343
B2/B3	50.0	68.4	48.9	​
L1	41.7	47.4	48.9	0.299
L2	16.7	31.6	11.1	​
L3	41.7	21.1	40.0	​
Disease duration (IQR), years	0.04 (2.74)	0.03 (1.93)	0.13 (3.17)	0.463
Receiving blood transfusion, %	91.7	78.9	71.1	0.135
Amount of blood transfusion (IQR), unit	4 (3)	4 (3)	5 (2)	0.792
Hb (IQR), g/dL	8.0 (2.0)	7.0 (1.3)	7.1 (2.6)	0.235
CRP (IQR), mg/L	49.14 (46.52)	31.00 (33.14)	35.32 (64.71)	0.288
ESR (IQR), mm/h	47^b^ (20)	41^b^ (33)	24^a^ (14)	**0.001**
CDAI (SD)	175.56^a^ (21.45)	178.65^a^ (22.83)	204.25^b^ (26.57)	**<0.001**
Treatment before bleeding, %	​	​	​	**0.019**
No treatment	16.7^a^	57.9^b^	48.9^b^	​
Infliximab therapy	16.7^a^	0.0^a^	6.7^a^	​
Non-biologic therapy	66.7^a^	42.1^a^	44.4^a^	​
Localized bleeding, %	58.3	73.7	51.1	0.247
Diffused bleeding, %	41.7	26.3	48.9	
Albumin (IQR), g/L	42.7 (8.4)	43.4 (8.1)	43.8 (9.4)	0.759

a, b: subsets with the same superscript letters did not differ significantly from each other (adjusted-*p* > 0.05), while differences between those with different superscript letters were significant (adjusted-*p* < 0.05). Non-biologic therapies included azathioprine, mesalazine, prednisone, salazosulfapyridine, and methylprednisolone. The bold values mean the p values were statistically significant.

For surgery and traditional groups, 57.9% and 48.9% of the respective patients did not receive any therapy before bleeding, while this value was only 16.7% for the infliximab group. Among the three groups, patients who had received infliximab treatment before bleeding were more likely to choose infliximab therapy directly in the event of acute SLGIB; however, patients who had not received any treatment before were more likely to choose surgery or traditional therapy (Table 1). All the patients in the infliximab group continued infliximab therapy after hemostasis, while only 31.6% of patients in the surgery and 35.6% of patients in the traditional therapy groups received infliximab therapy afterward (Table 2). None of the patients received surgery during the follow-up period.

**TABLE 2:** Outcomes and post-bleeding treatment in each group.

Variable	Infliximab therapy group	Surgery group	Traditional therapy group	*p*-value
CRP negative conversion rate, %	77.8^a^	23.5^b^	21.2^b^	**<0.001**
ESR negative conversion rate, %	71.4^a^	23.5^b^	19.4^b^	**0.002**
Increase in Hb level (SD), g/dL	2.0 (3.1)	2.8 (1.6)	2.2 (1.3)	0.298
Patients with complications after treatment	0.0^a^	78.9^b^	6.7^a^	**<0.001**
Total duration of hospitalization (IQR), days	7^a^ (5)	24^b^ (11)	11^a^ (5)	**<0.001**
Total cost of hospitalization (IQR), CNY	12,458.67^a^ (7,581.53)	59,764.65^b^ (24,256.54)	12,945.23^a^ (11,606.06)	**<0.001**
Treatment after bleeding, %	​	​	​	**<0.001**
No treatment	0.0^a^	31.6^b^	20.0^a,b^	​
Infliximab therapy	100.0^a^	31.6^b^	35.6^b^	​
Non-biologic therapy	0.0^a^	36.8^b^	44.4^b^	​

a, b: subsets with the same superscript letters did not differ significantly from each other (adjusted-*p* > 0.05), while differences between those with different superscript letters were significant (adjusted-*p* < 0.05). C-reactive protein (CRP), negative: < 5 mg/L. Erythrocyte sedimentation rate (ESR), negative: <20 mm/h for women; <15 mm/h for men. CNY, Chinese Yuan. Non-biologic therapies included azathioprine, mesalazine, prednisone, salazosulfapyridine, and methylprednisolone. The bold values mean the p values were statistically significant.

### Therapeutic efficacy of each group

In all three groups, the bleeding became unobvious within 1 week of treatment. The negative conversion rates of CRP and ESR were higher in the infliximab therapy group (77.8% for CRP and 71.4% for ESR) than the surgery (23.5% for CRP and 23.5% for ESR) and traditional therapy (21.2% for CRP and 19.4% for ESR) groups and were statistically significant (adjusted-*p* < 0.05 for both CRP and ESR). There was no significant difference in Hb level increase among the three groups (*p* = 0.298), implying that the three treatment types examined here have similar effects on Hb improvement (Table 2). In terms of post-treatment complications, the surgery group had the highest rate; there were no complications in any of the patients receiving infliximab therapy. In the surgery group, 15 patients reportedly showed intestinal fistula, intestinal obstruction, wound bleeding, or wound infection after treatment; furthermore, three patients in the traditional therapy group developed adverse reactions (two had interbowel abscess and one had intestinal fistula). The duration of hospitalization and associated costs were significantly lower in the infliximab therapy group than the surgery group (adjusted-*p* < 0.05) and similar in the traditional therapy group (adjusted-*p* > 0.05) (Table 2).

We also performed subgroup analysis for patients with localized and diffused bleeding. In the localized bleeding group, the negative conversion rates of CRP and ESR were higher for infliximab therapy but not significant given the limited sample sizes. The increase in Hb level remained similar among the three groups. In terms of complications as well as duration and cost of hospitalization, the results were similar to those in the general study population, with the surgery group having the highest values in these three aspects (Supplementary Table S1). In the diffused bleeding group, the outcomes were similar to the general results (Supplementary Table S2).

### Rebleeding risk of each group

**FIGURE 1:**
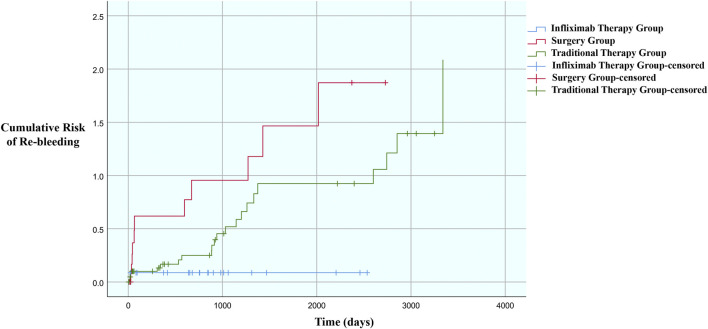
Cumulative risk of rebleeding in the different study groups.

During follow-up, rebleeding was reported by 36 patients (infliximab therapy: 2, surgery: 11, traditional therapy: 23). The rebleeding risk in the infliximab therapy group was significantly lower than those in the surgery (*p* = 0.001) and traditional therapy (*p* = 0.032) groups; however, the difference between the surgery and traditional therapy groups was not significant (*p* = 0.084) (Figure 1). To further explore the factors related to rebleeding, we performed univariate COX regression with the factors shown in Table 3 and included factors with *p* < 0.1 in the multivariate analysis. We found that the treatment for bleeding was an independent factor related to rebleeding; thus, compared to infliximab therapy, surgery [HR = 7.270, 95% CI: (1.574, 33.592), *p* = 0.011] and traditional therapy [HR = 4.395, 95% CI: (1.011, 19.113), *p* = 0.048] had relatively higher risk of rebleeding. Moreover, the Montreal classification of B1 was a predictive factor of rebleeding (Table 3). The localized/diffused bleeding status and albumin level are not related with rebleeding risk.

**TABLE 3:** Results of COX regression on potential factors related to rebleeding.

Parameter	Univariate analysis			Multivariate analysis		
	HR	95% CI	*p-*value	HR	95% CI	*p-*value
Age, year	0.995	[0.966, 1.025]	0.744	​	​	​
Male sex	1.623	[0.571, 4.615]	0.364	​	​	​
Smoking	1.862	[0.920, 3.768]	**0.084**	1.150	[0.553, 2.395]	0.708
With post-treatment complication	1.408	[0.655, 3.029]	0.381	​	​	​
Post-bleeding treatment						
No treatment	1	​	​	​	​	​
Infliximab therapy	0.958	[0.308, 2.979]	0.941	​	​	​
Non-biologic therapy	1.757	[0.589, 5.237]	0.312	​	​	​
Treatment for bleeding						
Infliximab therapy	1	​	​	1	​	​
Surgery	8.509	[1.878, 38.565]	**0.005**	7.270	[1.574, 33.592]	**0.011**
Traditional therapy	4.540	[1.053, 19.582]	**0.042**	4.395	[1.011, 19.113]	**0.048**
Montreal classification						
A1	1	​	​	​	​	​
A2	0.864	[0.367, 2.032]	0.737	​	​	​
A3	1.388	[0.432, 4.462]	0.582	​	​	​
B1	1	​	​	1	​	​
B2/B3	2.489	[1.187, 5.219]	**0.016**	2.202	[1.022, 4.746]	**0.044**
L1	1	​	​	​	​	​
L2	0.919	[0.306, 2.759]	0.880	​	​	​
L3	1.190	[0.573, 2.473]	0.641	​	​	​
Disease duration, years	0.927	[0.808, 1.063]	0.279	​	​	​
CDAI	0.993	[0.981, 1.004]	0.212	​	​	​
Receiving blood transfusion	0.603	[0.297, 1.224]	0.161	​	​	​
Localized bleeding	1	​	​	​	​	​
Diffused bleeding	0.567	[0.276, 1.166]	0.123	​	​	​
Albumin, g/L	1.009	[0.942, 1.080]	0.805	​	​	​

Non-biologic therapies included azathioprine, mesalazine, prednisone, salazosulfapyridine, and methylprednisolone. The bold values in the univariate analysis mean the p values were less than 0.1, which could be included in the multivariate analysis. The bold values in the multivariate analysis mean the p values were statistically significant.

## Discussion

In this study, we investigated the efficacies of different therapies for acute SLGIB in CD using a well-defined hospital-based inception cohort, with particular focus on whether infliximab therapy was more advantageous from the clinical and economic perspectives. To the best of our knowledge, this is a pilot effort on evaluating the efficacies, financial constraints, and prognosis of different therapies for acute SLGIB in CD. Our results demonstrate that infliximab therapy could achieve similar bleeding-healing effects as surgery and traditional therapy and that it may also offer anti-inflammatory advantages. With regard to patient safety, the surgery group showed the highest incidence rate of post-treatment complications among the three groups, while the post-treatment complication rates in the infliximab and traditional therapy groups were similar. In terms of the duration and costs of hospitalization, the surgery group had the highest values while the other two groups were similar and not significantly different. During follow-up, the infliximab therapy group reported the lowest risk of rebleeding; the COX regression analysis showed that surgery and traditional therapy could increase the risk of rebleeding compared to infliximab therapy.

In our study, the proportion of patients with CD of the ileum was the highest, which is related to the fact that inflammatory factors are more pronounced in the ileum. Studies have reported that creeping fat exists in patients with CD of the ileum (Atreya and Siegmund, 2021); as one of the sources of inflammation (Suau et al., 2022), creeping fat has garnered extensive attention in recent times. Studies have shown that patients with CD of the ileum require higher drug plasma concentrations to achieve mucosal healing (Takenaka et al., 2021) and that different lesion sites result in different manifestations and prognosis (Atreya and Siegmund, 2021). In the present study, the location of CD did not influence the risk of rebleeding.

The incidence of post-treatment complications was highest in the surgery group. We suggest that this could be related to the release of local inflammatory factors, intestinal anatomy changes after surgery, and proficiency of the surgeon. The CD lesions show segmental distribution. Patients with acute SLGIB are more prone to multiple deep ulcers such that extensive bowel resection could cause short bowel syndrome. Moreover, the highly inflammatory state of the intestine itself is not conducive to postoperative anastomotic healing, which is more likely to cause postoperative intestinal stenosis and intestinal fistula. A study conducted in Japan on 1,871 CD patients who received surgery reported that the 5- and 10-year reoperation rates were 23.4% and 48.0%, respectively; perianal disease and ileocolic CD could increase the risk of reoperation, while anti-TNF therapy after surgery could decrease this risk (Shinagawa et al., 2020). Another research from Denmark showed similar results among 631 initially resected patients, with the 1-, 5-, and 10-year reoperation rates being 12.6%, 22.4%, and 32.2%, respectively, and the main reasons for reoperation being disease activity and stoma reversal (Poulsen et al., 2024).

The rebleeding risk in the infliximab group was significantly lower than those in the surgery and traditional therapy groups. This is attributable to the inhibition of inflammatory reactions in the infliximab therapy group that disrupts the development of CD. The main feature of CD is inflammation of the gastrointestinal tract resulting from the interactions among genes, gut microbiota, and environmental factors (Narula et al., 2023). Traditional medications like somatostatins or octreotide can only control bleeding symptomatically and temporarily. However, infliximab therapy can inhibit immunity, reducing infiltration by local inflammatory factors and promoting rapid mucosal healing (Baumgart and Le Berre, 2021). We also noted that patients with B1 Montreal classification were less likely to rebleed; the reason for this could be that B1 in the Montreal classification implies a non-narrow and non-penetrating condition, which indicates a less-severe disease status.

Despite these findings, there remained a small proportion of patients who experienced rebleeding after infliximab therapy, which could be related to high drug clearance and insufficient drug exposure; in this regard, Gibson et al. (2015) considered the concept of accelerated infliximab induction treatment. For acute severe ulcerative colitis patients with poor drug response, Gibson et al. (2020) reported the use of accelerated dosing of infliximab. Accelerated infliximab induction was also reported for the treatment of CD with acute SLGIB (Zeng et al., 2022), so it may be applicable in CD patients with acute SLGIB showing poor initial responses. Aside from traditional hemostatic therapy, surgery, and infliximab, interventional treatment for bleeding is also developing rapidly at present. However, the diverse lesion sites of CD in patients mean that there may be more than one potential bleeding location, which is a challenge when selecting the embolization site. In transarterial embolization for acute lower gastrointestinal bleeding, there is still a risk of rebleeding (20.7%) and post-embolization ischemia (7.5%) (Yu et al., 2024).

We explored published English literature and found 17 cases of CD-related acute SLGIB from nine studies that reported treatment with infliximab (Zeng et al., 2022; Akazawa et al., 2014; Ando et al., 2009; Aniwan et al., 2012; Belaiche and Louis, 2002; Cunningham et al., 2014; Meyer and Levine, 2009; Papi et al., 2003; Tsujikawa et al., 2004). Of these patients, eight were male and the remaining nine were female, and their mean age was 41.59 ± 23.93 years. None of these patients had received infliximab treatment before. Most of the patients showed multiple potential bleeding sites, of which the ileum (n = 7) and ileocolon (n = 7) were the most common disease locations (Zeng et al., 2022; Akazawa et al., 2014; Ando et al., 2009; Aniwan et al., 2012; Belaiche and Louis, 2002; Cunningham et al., 2014; Meyer and Levine, 2009; Papi et al., 2003; Tsujikawa et al., 2004). Three of these patients received surgery but did not show significantly improved hematochezia, and two of them had undergone multiple surgeries. All patients received infliximab therapy when the conventional treatments and surgery could not stanch the bleeding effectively. Eight patients achieved the goal of hemostasis within 1 week and received only one course of infliximab treatment. Four of the patients had metastasized on the day of first infliximab therapy but still finished the second course. One patient experienced bleeding stoppage with accelerated infliximab induction; two patients achieved hemostasis after approximately 2 weeks of infliximab therapy, and five patients received long-term maintenance therapy (Supplementary Table S3).

Our study has some notable limitations, including the limited number of cases, which could affect the reliability of the results. In addition, the grouping of cases into different treatment plans was not random but rather determined based on patient preferences and clinical evaluations. Lastly, this study was conducted retrospectively and is therefore prone to bias from unrecognized or unmeasured factors, such as recall bias. Nevertheless, the strength of our present study is that we report and utilize a well-defined inception cohort, which enables us to evaluate the efficacies and economic issues associated with infliximab therapy, surgery, and conventional therapy for CD patients with acute SLGIB; we also conducted long-term follow-up on the patients. The present work is a multicenter study conducted in Hunan Province of China, and our results are consistent with the findings of a previous study by Kim et al. (2021). The earlier study only analyzed the efficacy of hemostasis and rebleeding risk, whereas our research additionally compares Hb level improvements, anti-inflammatory effects, and rate of complications, along with the duration and cost of hospitalization among the different groups.

Our findings suggest that infliximab therapy is an effective method for controlling acute SLGIB in CD patients and that it may be better than surgery and traditional treatment in terms of anti-inflammatory effects and rebleeding prevention. Furthermore, the cost and duration of hospitalization in the infliximab therapy group are similar to those in the traditional treatment group and lower than those in the surgery group. In conclusion, infliximab therapy should be considered in clinical practice for acute SLGIB in CD to achieve better prognosis and economic benefits.

## Data Availability

The raw data supporting the conclusions of this article will be made available by the authors without undue reservation.
