# Supplementary material for: Different treatments for Crohn’s disease complicated by severe acute lower gastrointestinal bleeding: infliximab therapy is critical and cannot be ignored
Source: Front Pharmacol. 2026 Feb 23;17:1687439. doi: 10.3389/fphar.2026.1687439 (PMC12967973; doi:10.3389/fphar.2026.1687439)
Supplement: Supplementary file 1 [file Supplementaryfile1.docx]

Supplemental Table 1. Outcomes and post-bleeding treatment in localized bleeding subgroup

| Variable | Infliximab Therapy Group | Surgery Group | Traditional Therapy Group | *p* value |
| --- | --- | --- | --- | --- |
| CRP negative conversion rate, %  ESR negative conversion rate, %  The raise of Hb level (SD), g/dl | 55.6  71.4  1.1 (3.2) | 30.8  30.8  2.7 (1.4) | 17.6  22.2  2.5 (1.3) | 0.142  0.072  0.235 |
| Patients with complication after treatment, %  Total length of hospital stay (IQR), day | 0.0^a^  8^a^ (4) | 71.4^b^  21^b^ (12) | 8.7^a^  11^a^ (5) | **<0.001**  **<0.001** |
| Total hospitalization costs (IQR), CNY | 12154.88^a^ (5659.84) | 59764.65^b^ (21748.72) | 9128.57^a^ (10459.71) | **<0.001** |
| Treatment after bleeding, % |  |  |  | **<0.001** |
| No treatment | 0.0^a^ | 35.7^b^ | 21.7^a, b^ |  |
| Infliximab therapy | 100.0^a^ | 28.6^b^ | 26.1^b^ |  |
| Non-biologic therapy | 0.0^a^ | 35.7^b^ | 52.2^b^ |  |

a, b: Subsets with the same subscript letter do not differ significantly from each other (adjusted-*p*>0.05), while the differences between those with different subscript letters are significant (adjusted-*p*<0.05). CRP negative: < 5mg/L. ESR negative: < 20mm/hr for female, < 15mm/hr for male. CNY: China Yuan. Non-biologic therapy included azathioprine, mesalazine, prednisone, salazosulfapyridine, or methylprednisolone etc. The bold values mean the *p* values were statistically significant.

Supplemental Table 2. Outcomes and post-bleeding treatment in diffused bleeding subgroup

| Variable | Infliximab Therapy Group | Surgery Group | Traditional Therapy Group | *p* value |
| --- | --- | --- | --- | --- |
| CRP negative conversion rate, %  ESR negative conversion rate, %  The raise of Hb level (SD), g/dl | 100.0^a^  71.4^a^  3.2 (2.7) | 0.0^b^  0.0^a, b^  3.0 (2.3) | 25.0^b^  15.4^b^  1.8 (1.2) | **<0.001**  **0.012**  0.277 |
| Patients with complication after treatment, %  Total length of hospital stay (SD), day | 0.0^a^  8^a^ (6) | 100.0^b^  26^b^ (4) | 4.5^a^  10^a^ (4) | **<0.001**  **<0.001** |
| Total hospitalization costs (IQR), CNY | 13958.10^a^ (9587.60) | 52032.99^b^ (36849.89) | 15490.94^a^ (13675.44) | **0.011** |
| Treatment after bleeding, % |  |  |  | **0.030** |
| No treatment | 0.0^a^ | 20.0^a^ | 18.2^a^ |  |
| Infliximab therapy | 100.0^a^ | 40.0^b^ | 45.5^b^ |  |
| Non-biologic therapy | 0.0^a^ | 40.0^a^ | 36.4^a^ |  |

a, b: Subsets with the same subscript letter do not differ significantly from each other (adjusted-*p*>0.05), while the differences between those with different subscript letters are significant (adjusted-*p*<0.05). CRP negative: < 5mg/L. ESR negative: < 20mm/hr for female, < 15mm/hr for male. CNY: China Yuan. Non-biologic therapy included azathioprine, mesalazine, prednisone, salazosulfapyridine, or methylprednisolone etc. The bold values mean the *p* values were statistically significant.

Supplemental Table 3. Successful control of severe lower gastrointestinal bleeding in Crohn's disease with Infliximab therapy.

| Author | Number | Age (year) | Sex | Duration of CD | Initial Treatment | Hb (g/dL) | Location of CD | Infliximab Therapy | Bleeding controlled in |
| --- | --- | --- | --- | --- | --- | --- | --- | --- | --- |
| Belaiche (AJG 2002) | 1 | 28 | Female | 3 years | Blood transfusion (10 units) + Budesonide (9mg/d) + AZA (125mg/d) | 7.6 | Ileocolon | Infliximab, 5mg/kg (day 0, week 2) | 14 days |
|  | 2 | 59 | Female | 9 years | Blood transfusion (4 units) + Oral steroids + metronidazole + ciprofloxacine + AZA (100mg/d) | 8 | Colon | Infliximab, 5mg/kg (day 0) | 4 days |
| Papi (J Clin Gastroenterol 2003) | 1 | 50 | Male | 9 months | Surgery (1 time) + prednisolone(1mg/kg/d) + AZA (2mg/kg/d) | 9 | Ileum | Infliximab, 5mg/kg (day 0, week 2, week 6) | NA |
|  | 2 | 68 | Male | 34 years | Blood transfusion (4 units) + Surgery (3 times) + mesalazine (2.4g/d) + AZA (2mg/kg/d) | 4.4 | Ileocolon | Infliximab, 5mg/kg (day 0) | NA |
| Tsujikawa (J Gastroenterol 2004) | 1 | 31 | Male | 12 years | Surgery (2 times) + salazosulfapyridine | 13.2 | Ileocolon | Infliximab, 5mg/kg (day 0, week 2, week 6) | NA |
| Ando (Inflamm Bowel Dis 2009) | 1 | 16 | Female | 1 year | Blood transfusion (6 units) + Prednisolone (40mg/d) + mesalazine (3g/d) | 12 to 5.2 | Colon | Infliximab, 5mg/kg (day 0) | 3 days |
| Meyer (Inflamm Bowel Dis 2009) | 1 | 19 | Female | 6 years | Blood transfusion (4 units) + Prednisolone + mesalazine | 9.1 to 6.8 | Ileocolon | Infliximab, 5mg/kg (day 0, week 2, week 6) | NA |
| Aniwan (World J Gastroenterol 2012) | 1 | 11 | Female | 2 months | Blood transfusion (4 units) + Prednisolone (35mg/d) + AZA (1.5mg/kg/d) | 11 to 8 | Colon | Infliximab, 5mg/kg (day 0) | 1 day |
|  | 2 | 19 | Female | 7 months | Blood transfusion (1 unit) + Budesonide (9mg/d) + mesalazine (2g/d) | 12 to 10 | Ileocolon | Infliximab, 5mg/kg (day 0, day 10) | 10 days |
|  | 3 | 59 | Male | 0 day | Blood transfusion (6 units) | 10 to 8 | Ileum | Infliximab, 5mg/kg (day 0, week 2) | 1 day |
|  | 4 | 86 | Male | 0 day | Blood transfusion (7 units) | 12 to 8.5 | Ileum | Infliximab, 5mg/kg (day 0, week 2) | 1 day |
|  | 5 | 71 | Female | 0 day | Blood transfusion (3 units) | 13 to 10 | Ileum | Infliximab, 5mg/kg (day 0, week 2) | 1 day |
|  | 6 | 50 | Female | 0 day | Blood transfusion (2 units) | 14 to 10 | Ileum and jejunum | Infliximab, 5mg/kg (day 0, week 2) | 1 day |
|  | 7 | 71 | Male | 0 day | Blood transfusion (6 units) | 11 to 6.5 | Ileum | Infliximab, 5mg/kg (day 0) | 1 day |
| Akazawa (BMC Research Notes 2014) | 1 | 26 | Male | 1 month | Blood infusion + Prednisolone (50mg/d) + mesalazine | 5.5 | Ileocolon | Infliximab, 250 mg (day 0, week 2, week 6) | NA |
| Cunningham  (BMJ Case Rep 2014) | 1 | 27 | Female | NA | Blood transfusion + Intravenous hydrocortisone + ciprofloxacin + metronidazole | NA | Ileum | Standard induction and maintenance  infliximab | NA |
| Zeng (World J Clin Cases 2022) | 1 | 16 | Male | 0 day | Methylprednisolone (40mg/d) | 11.2 to 8 | Ileocolon | Infliximab, 5mg/kg (day 0, day 7) | NA |

CD: Crohn's disease; Hb: Hemoglobin; AZA: Azathioprine
